# Complete mitogenome of the chlorophyte green alga *Marsupiomonas* sp. NIES 1824 (Pedinophyceae)

**DOI:** 10.1080/23802359.2019.1710283

**Published:** 2020-01-14

**Authors:** Monique Turmel, Christian Otis, Claude Lemieux

**Affiliations:** Département de biochimie, de microbiologie et de bio-informatique, Institut de Biologie Intégrative et des Systèmes, Université Laval, Québec, Canada

**Keywords:** Chlorophyta, variant genetic code, fragmented rnl gene, Pedinophyceae, phylogenomics

## Abstract

The 25,137-bp mitogenome of the green alga *Pedinomonas minor* (Pedinomonadales, Pedinophyceae), which belongs to a basal class of the core Chlorophyta, is unusual in displaying a reduced gene content as well as other derived traits. Here, we present the mitogenome of *Marsupiomonas* sp. NIES 1824 (Marsupiomonadales, Pedinophyceae). Despite its smaller size, this 24,252-bp genome encodes twice as many genes (39) as its *P*. *minor* homolog. Besides gradual gene erosion, our comparative analyses revealed that major changes in GC content and codon usage led to the gain of distinct, noncanonical genetic codes during evolution of the mitogenome in the Pedinophyceae.

The Pedinophyceae is a small class of green algae belonging to the Chlorophyta that comprises planktonic and terrestrial uniflagellates (Moestrup 1991). The mitogenome of *Pedinomonas minor* (Pedinomonadales) is currently the only one available for this class (Turmel et al. 1999). In several respects, including gene content and rRNA gene organization, this 25,137-bp genome, which encodes only 21 genes compared to the 61–88 found in prasinophyte mitogenomes with an ancestral pattern of evolution (Turmel et al. 1999; Robbens et al. 2007; Hrda et al. 2017; Satjarak et al. 2017; Turmel, Lopes Dos Santos, et al. 2019; Turmel, Otis, et al. 2019), resembles the reduced mitogenomes of chlamydomonadalean algae (Chlorophyceae) (Turmel et al. 1999), although no specific phylogenetic affinities exist between the Pedinophyceae and Chlamydomonadales (Lemieux et al. 2014; Turmel et al. 2016). Here we present the mitogenome of *Marsupiomonas* sp. NIES 1824 (Marsupiomonadales, Pedinophyceae).

This strain was obtained from the Microbial Culture Collection at the National Institute for Environmental Studies (Tsukuba, Japan). Fragments of 700 bp derived from a A + T-rich DNA fraction were sequenced by the Genomic Analysis Platform of Laval University using 454 pyrosequencing. The methodologies used for DNA isolation, library construction, read assembly, and gene annotation are described in (Lemieux et al. 2014).

The *Marsupiomonas* mitogenome is a circular molecule of 24,252 bp (GenBank MN782006) with an overall GC content of 51.4%, a higher value compared to that observed for *P*. *minor* (22.2%). Although it is smaller than its *P*. *minor* homolog, its gene repertoire is about twice as large, comprising two rRNA genes, 20 tRNA genes (instead of eight in *P*. *minor*) and 17 protein-coding genes (instead of 11). *Marsupiomonas* is missing *atp8*, *trnL*(caa) and *trnW*(uca) (the latter gene product enables decoding of UGA as tryptophan) relative to *P*. *minor*, while the later alga is missing seven protein-coding and 14 tRNA genes relative to *Marsupiomonas*. The *rnl* gene occurs as two separate pieces in both algae and features the same fragmentation site. All *Marsupiomonas* genes are intronless and like their *P*. *minor* homologs are encoded on the same DNA strand although gene order is extremely scrambled.

Analyses of codon usage unveiled important differences between the two pedinophycean mitogenomes. *Marsupiomonas* protein-coding genes are characterized by a stronger bias toward GC-rich codons, the absence of UGA codon reassignment to tryptophan, the lack of UUR codons and a disproportionally higher frequency of AGR codons. Our protein sequence alignments predict that AGR codons are decoded as alanine instead of arginine as recently reported for mitogenomes of the Sphaeropleales (Chlorophyceae) (Noutahi et al. 2019; Zihala and Elias 2019).

Phylogenetic analysis of concatenated proteins using RAxML v.8.2.3 (Stamatakis 2014) recovered the two pedinophyceans as a weakly supported clade displaying very long branches within the core Chlorophyta (Figure 1).

**Figure 1.**
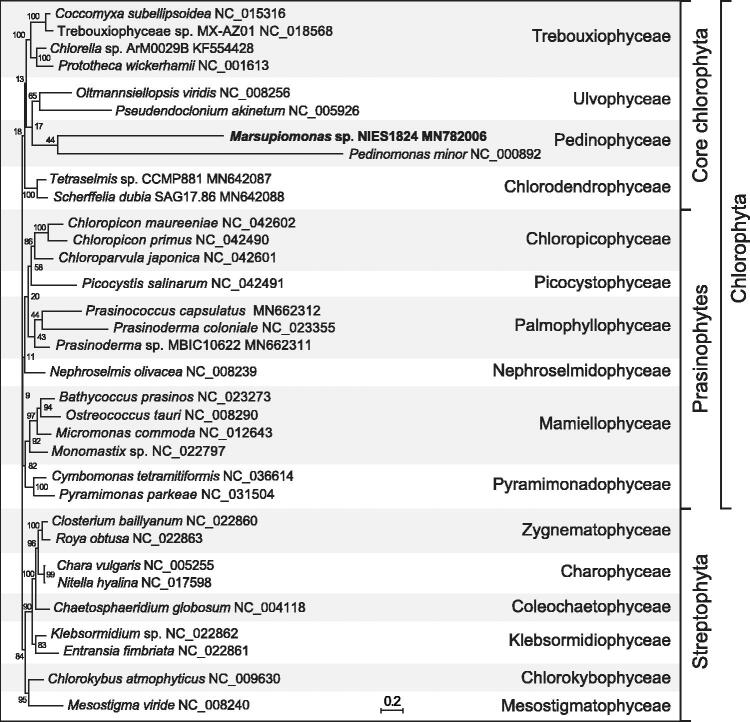
RAxML analysis of 16 concatenated mitogenome-encoded proteins from 24 chlorophytes and nine streptophyte green algae. The best-scoring tree is shown, with the bootstrap support values (100 replicates) reported on the nodes. GenBank accession numbers are provided for the mitogenomes of all taxa. The scale bar denotes the estimated number of amino acid substitutions per site. The data set was generated using the predicted protein sequences derived from the following genes: *atp6, 9, cob, cox1, 2, 3, nad1, 2, 3, 4, 4 L, 5, 6, rpl6, 16, rps12*. Following alignment of the sequences of individual proteins with Muscle v3.7 (Edgar 2004), ambiguously aligned regions were removed using TrimAL v1.4 (Capella-Gutierrez et al. 2009) with the options block = 6, gt = 0.7, st = 0.005 and sw = 3, and the protein alignments were concatenated using Phyutility v2.2.6 (Smith and Dunn 2008). The phylogenetic analysis was carried out under the LG4X model (Le et al. 2012).

Overall, our results indicate that the common ancestor of the Pedinomonadales and Marsupiomonadales possessed a fragmented *rnl* gene and a repertoire of 42 genes. Gene erosion thus occurred stepwise during the evolution of the Pedinophyceae and was accompanied by the acquisition of noncanonical genetic codes.
